# Impact of proximity of thermoelectric power plants on bronchial obstructive crisis rates

**DOI:** 10.1186/s12889-016-4008-7

**Published:** 2017-01-19

**Authors:** Tamara Ugarte-Avilés, Carlos Manterola, Ricardo Cartes-Velásquez, Tamara Otzen

**Affiliations:** 1 Medical Sciences Programs, Universidad de La Frontera, Temuco, Chile; 2 Facultad de Medicina, Universidad de Concepción, Concepción, Chile; 3 Grupo de Investigación en Salud Cardiovascular y Respiratoria (IDEAS-CVR), Concepción, Chile; 4 Center for Biomedical Research, Universidad Autónoma de Chile, Temuco, Chile; 5 Facultad de Odontología, Universidad de Concepción, Concepción, Chile; 6 Facultad de Ciencias de La Salud, Universidad de Tarapacá, Arica, Chile; 7 Universidad Científica del Sur, Chorrillos, Peru; 8 Universidad Autónoma de Chile, Temuco, Chile

**Keywords:** “Environmental Pollution” [Mesh], “Power Plants” [Mesh], “Airway Obstruction” [Mesh], Thermoelectric Power Plants, “Chile” [Mesh]

## Abstract

**Background:**

Environmental pollution is a risk factor for cardiorespiratory diseases. Energy generated by thermoelectric power plants (TEPP) represents a relevant source of pollution. The aim of this study was to evaluate the relationship between living near a coal-fired TEPP and the consultation rates for bronchial obstructive crises (BOC) in the province of Concepción, Chile.

**Methods:**

Population-based study. The epidemiological weeks from 2012 to 2014 were analyzed. The dependent variable was the emergency consultation rate for BOC in two health centers within 5 km of a TEPP (Coronel) and two that were more than 40 Km away from a TEPP (Talcahuano). The independent variables were the commune, climatological variables (air temperature and relative atmospheric humidity), environmental pollutants (PM_10_, PM_2.5_ and nitrogen oxide), weeks with the highest consultation rate and the years. Rates, Pearson’s correlation and gross risk measures were calculated and adjusted for environmental and climatological variables.

**Results:**

BOC rates were significantly higher in Coronel (RR = 4.9 95% CI 4.0–5.8; *p* < 0.05). The PM_2.5_ it showed the strongest correlation with BOC rates (*r* = 0.3; *p* < 0.01) in Coronel, but not Talcahuano. Linear regression modelling indicated that proximity to a TEPP (health center location) and temperature explained 26 and 18% of the variance in BOC rates, respectively.

**Conclusions:**

Rates of emergency consultation for BOC were significantly higher among a population living within 5 km of a coal-fired TEPP than those living 40 km away.

## Background

According to the World Health Organization, environmental pollution is one of the greatest global health risks, causing more than 7 million premature deaths per year [1].

Non-natural sources that emit pollutants into the atmosphere can vary significantly between cities, but to a large extent they come from the use of fossil fuels in various daily activities, such as domestic heating, industry, use of motorized vehicles, smelting, incineration and power generation, as is the case with thermoelectric power plants (TEPP) [2, 3].

A TEPP produces electrical energy from the combustion of fossil fuels like coal, oil and natural gas. The main emissions released into the atmosphere are sulfur dioxide (SO_2_), nitrogen oxide (NO), particulate matter (PM), carbon monoxide (CO) and greenhouse gases like carbon dioxide (CO_2_) [4].

Studies conducted in several countries have provided evidence of the link between atmospheric pollutants and the increase in emergency consultations for chronic respiratory diseases (CRD) like asthma and chronic obstructive pulmonary disease (COPD) [5, 6–8]. Asthma is one of the diseases with the highest morbidity in all age groups and economic strata; it has no age of presentation and is associated with psychogenic and environmental components. The latter include pollutants like ozone, nitrogen and sulfur dioxide, causing mainly bronchoconstriction and increase in the production of bronchial secretions [9].

COPD is characterized by a restricted airflow that is not totally reversible, usually progressive and associated with inflammation in the lungs in response to gases or toxic particles [10].

Few studies have sought the link between environmental pollution by TEPP and CRD among the population, specifically during periods of exacerbation or bronchial obstructive crisis (BOC).

This article was written following the MInCir initiative for the reporting of descriptive observational studies [11].

The aim of this study was to identify the association between living near a coal-fired TEPP and the consultation rates for BOC in the 2012–2014 period in the province of Concepción, Bío-Bío Region, Chile.

## Methods

### Design

Ecological population-based study.

### Scenario

Emergency services of four Family Health Centers (CESFAM) in the province of Concepción: CESFAM Yobilo, CESFAM Lagunilla, CESFAM Los Cerros and CESFAM San Vicente. CESFAM are public health centers providing primary care. In some cases they have emergency primary care units (SAPU, in Spanish) that attend emergency cases, including BOC.

### Participants

Consultations for BOC obtained from the previously indicated SAPU: two in the commune of Coronel (near a TEPP), and two in the commune of Talcahuano (more than 40 Km away from a TEPP). The number of weekly consultations was obtained from the Office of Health Statistics and Information (DEIS, in Spanish) for the 2012–2014 period. As a unit of analysis, the epidemiological weeks from 2012, 2013 and 2014 were included. One hundred fifty-six weeks total.

### Inclusion and exclusion criteria

Were included two Health centers near to a conventional coal-fired TEPP were included, specifically no more than 5 km around their area of coverage; and also two health centers located more than 40 km from a TEPP. These health centers had to be located in areas of similar climatological and sociodemographic characteristics, both areas are industrial places and also have emergency care statistics in the DEIS records from 2011 onwards.

### Sampling

The centers were chosen by convenience, respecting the inclusion criteria. All the centers in the province were assessed and these four ultimately fulfilled the established criteria.

### Variables

The dependent variable was the emergency consultation rate for BOC, obtained by the number of consultations recorded in the DEIS and equivalent to the pathologies between J40 and J46 in the International Classification of Diseases (ICD-10), i.e., chronic bronchitis, emphysema, asthma, status asthmaticus, bronchiectasis and other COPD. The independent variables considered were: climatological variables (air temperature measured in degrees Celsius, and relative atmospheric humidity measured in percentage); presence of environmental pollutants (PM_10_, PM_2.5_ and nitrogen oxide, measured in μg/m^3^); presence/absence of TEPP (Coronel/Talcahuano); years and weeks with the highest consultation rate. The latter variable was included to explore if the association of the rates with the independent variables was similar to the presented in the rest of the epidemiological weeks.

### Data collection

The data about the climatic variables in Coronel were obtained from the National Air Quality System (SINCA, in Spanish) of the Chilean Ministry of the Environment, specifically from the weather station in north Coronel. In Talcahuano data were obtained from the Chilean Navy’s Weather Center. The environmental pollutants were obtained from SINCA as weekly average concentration. In Coronel from the Cerro Merquén station and South Coronel station, and in Talcahuano from the Nueva Libertad station. The population legitimately enrolled in each municipal health center was obtained from the Office of Health Administration (DAS, in Spanish) of Talcahuano and the DAS of the Municipality of Coronel.

### Statistics

The consultation rates for BOC were calculated for the health centers per 1000 inhabitants, using the number of consultations as the numerator and the population attended by the health centers as the denominator. These calculations were made generally for both places and independently for Talcahuano and Coronel, and they were also made for the full period (2012–2014) and for each year independently. In addition, the rates were calculated for each of the epidemiological weeks between 2012 and 2014 in Talcahuano and Coronel. Then, descriptive statistics were applied, including mean ± standard deviation (SD); mean differences of the climatological variables and the environmental pollutants between places were identified (Coronel/Talcahuano). Mean differences of the rates of BOC were calculated between places using a student’s *t*-test for independent samples. Then, the association between consultation rates for BOC, environmental pollutants and climatic factors were identified according to place and years using Pearson’s correlation. The ratios of the means of the weekly consultation rates for BOC of the places with and without TEPP were calculated; and finally, simple and multiple linear regressions models were used to determine the association between BOC rates, considering the rate as a dependent variable, and all the independent variables of the study. The variables selected to enter the regression analyses were those that correlated significantly with the BOC rates. All the analyses considered 95% confidence intervals using the statistical program IBM SPSS 20 and Microsoft Excel 2011, v. 14.0.0 to create the graphs.

### Ethical principles

Considering that this study worked with secondary data collected from various sources (DEIS, SINCA), the formal authorization of the people in the process was not required. Nevertheless, it was authorized by the Research and Bioethics Committee of the Faculty of Medicine at the University of Concepción, as well as by the DAS in Talcahuano and Coronel.

## Results

According to data contributed by the DAS in Coronel in 2012 there were 53,229 people registered in the participating health centers, 58,170 in 2013 and 56,788 in 2014, of which 46.77% were men and 20.18% were under 14 years. In the commune of Talcahuano there were 55,551 people registered in 2012, 57,939 in 2013 and 56,424 in 2014, of which 48.13% were men and 21.18% were under 14 years.

For the 2012–2014 period there were 5162 consultations for BOC in Coronel and 2458 in Talcahuano. Specifically in Coronel in 2012 there were 1845 consultations, in 2013 and 2014 there were 1744 and 1573 consultations respectively. In Talcahuano in 2012 there were 490 consultations, in 2013 and 2014 there were 833 and 1135 consultations respectively.

The PM_10_, PM_2.5_ and NO averages for Coronel and Talcahuano are shown in the Table 1. When the average of temperature was compared, statistically significant differences were not observed between Coronel and Talcahuano. The average temperature of Coronel during the 3 years was of 13.3 °C, (maximum, 19.03 °C and minimum, 7.48 °C). In Talcahuano the average temperature was 13.68 °C, (maximum, 18.8 °C and minimum, 8.6 °C).

**Table 1:** Mean (+/− SD) concentration of air pollutants detected in two towns in the province of Concepcion, Chile. 2012–2014

Year	Pollutant	Commune	Mean ± SD	*t*	*p*	95% CI
The entire study period	PM_2.5_	Talcahuano	19.7 ± 6.7	6.0	<0.01	3.9; 7.8
		Coronel	14.8 ± 9.2			
	PM_10_	Talcahuano	58.3 ± 16.1	−1.2	0.2	−7.7; 1.8
		Coronel	62.2 ± 24.2			
	NO	Talcahuano	9.6 ± 9.5	−6.1	<0.01	−9.5; −4.9
		Coronel	16.4 ± 9.7			

**Fig. 1:**
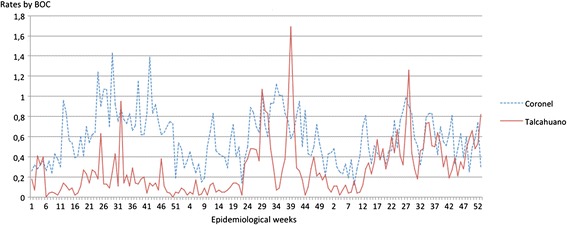
Graph of consultation rates by BOC for epidemiological weeks for the period 2012–2014, in Coronel and Talcahuano

In Coronel the average total weekly consultation rate for BOC for the 3 years was 0.6 ± 0.3SD per 1000 inhabitants with a maximum of 1.4 recorded in week 29 of 2012, between July 16 and 22. The minimum was recorded in week 9 of 2014, between February 24 and March 2, with a rate of 0.1 consultations per 1000 inhabitants. The average total weekly consultation rate for BOC for the 3 years for the commune of Talcahuano was 0.3 ± 0.3 SD per 1000 inhabitants with a maximum of 1.7 recorded in week 39 of 2013, between September 23 and 29. The observed minimum was zero consultations and this was recorded in weeks 6 and 50 of 2012, between February 6 and 12 and December 10 and 16, respectively (Fig. 1). The comparison of BOC rates of Coronel and Talcahuano for every single year and whole period are shown in Table 2.

**Table 2:** Mean (+/− SD) bronchial obstructive crisis (BOC) rates recorded in hospital records for two towns in the province of Concepcion, Chile. 2012–2014

Year	Rate (×1000 inhabitants)	Commune	Mean ± SD	*t*	*p*	95% CI
All	BOC	Talcahuano	0.3 ± 0.3	−10.5	<0.01	−0.3; −0.2
		Coronel	0.6 ± 0.3			
2012	BOC	Talcahuano	0.2 ± 0.2	−10.6	<0.01	−0.4; −0.6
		Coronel	0.7 ± 0.3			
2013	BOC	Talcahuano	0.3 ± 0.3	−5.3	<0.01	−0.2; −0.4
		Coronel	0.6 ± 0.3			
2014	BOC	Talcahuano	0.4 ± 0.2	−3.2	<0.01	−0.1; −0.2
		Coronel	0.5 ± 0.2			

The rate ratio was 4.9 (95% IC 4.0; 5.8) for the whole period, i.e., the consultations for BOC in Coronel were 4.9 times higher than in Talcahuano. The consultations for BOC in Coronel were higher within each of the 3 years with a RR of 6.7 (95% CI 5.2; 8.1), 5.7 (95% CI 3.7; 7.7) and 2.4 (95% CI 1.7; 3.1) for years 2012, 2013 and 2014 respectively.

In the analysis of the correlations for each commune, we found that in Talcahuano only the temperature presented a significant correlation with *p < 0.001* and a moderate and indirect intensity of r −0.4, indicating that the lower the temperatures the higher the rates, and vice versa. None of the remaining variables showed a significant correlation (Table 3).

**Table 3:** Pearson’s correlation between bronchial obstructive crisis (BOC) rates recorded in hospital records and pollution and weather records for two towns in the province of Concepcion, Chile. 2012-2014

	Temperature		Relative humidity		PM_2.5_		PM_10_		NO	
	Coronel	Thno	Coronel	Thno	Coronel	Thno	Coronel	Thno	Coronel	Thno
Consultation rate for BOC	−0.5 ^*^	−0.4 ^**^	0.3 ^**^	0.03	0.3 ^**^	0.2	0.2 ^**^	−0.9	0.1	0.0

*Thno* Talcahuano

^*^ *p* value < 0.05

^**^ *p* value < 0.01

In Coronel the situation was very different. Here all the variables, except NO, were significantly correlated with the rates with values of *p < 0.01*. The temperature had the greatest intensity (Table 3).

Finally, the regression analysis revealed that in the simple linear regression, when each independent variable was analyzed separately, two contributed to the variance with significant percentages. These were temperature, with a corrected R^2^ of 0.18, explaining 18.0% of the variance, and place, with a R^2^ of 0.26, explaining 26.0% of the variance. When the β value was analyzed, we observed that the temperature presents a β of −0.05, i.e., the lower the temperature the higher the consultation rate for BOC (Table 4).

**Table 4:** Simple linear regression between bronchial obstructive crisis (BOC) rates recorded in hospital records and pollution and weather records for two towns in the province of Concepcion, Chile. 2012–2014

Independent variable	F	*p*	Corrected R^2^	β	Error	*p*
Place	111.2	<0.000	0.26	0.31	0.03	<0.000
Temperature	65.3	<0.000	0.18	−0.05	0.00	<0.000
Humidity	2.3	0.13	0.004	−0.00	0.00	0.12
PM_10_	3.8	0.05	0.01	0.00	0.00	0.05
PM_2.5_	0.1	0.76	−0.001	−0.00	0.00	0.76
NO	15.7	0.00	0.05	0.00	0.00	<0.000
Independent variable^a^	F	*p*	Corrected R^2^	β	Error	*p*
Place	23.3	<0.000	0.28	0.41	0.09	<0.000
Temperature	1.1	0.30	0.00	−0.02	0.03	0.30
Humidity	0.0	0.90	−0.02	0.00	0.00	0.90
PM10	3.3	0.08	0.04	0.00	0.00	0.08
PM2.5	0.5	0.47	−0.00	−0.00	0.00	0.47
NO	0.4	0.54	−0.01	0.00	0.00	0.54

^a^Weeks with highest consultation rates

In order to examine what occurred in the weeks with the most frequent consultation for BOC, the 30 weeks of greatest consultation (10 per year) were analyzed. In this analysis, it was verified that only the place has an effect (Table 4).

The multiple linear regression analysis was tested with several models; we found that the model that explained the highest percentage of the variance included only the place and the temperature as confounding variables. The corrected R^2^ of the model was 0.44, explaining 44.0% of the variance. The β values of both variables, 0.31 (*p* < 0.000) to place and −0.05 (*p* < 0.000) to temperature, indicated that a lower temperature and living in Coronel were associated with higher consultation rates for BOC.

## Discussion

The consultation rate for BOC presented a RR of 4.9 between Coronel and Talcahuano. In the linear regression model we observed that both in the initial analysis and in the one of the weeks with the highest consultation rates, the variable place explained the greatest percentage of variance, reaffirming the hypothesis that living in the neighborhoods of a coal-fired TEPP increases the risk of consulting for certain respiratory pathologies, in this case, BOC.

The specific and isolated impact of environmental pollution caused by TEPP is not completely measurable. All the studies conducted on this subject have been ecological, population-based in nature and in none of them has it been possible to isolate in any comprehensive way the pollution originating specifically from the TEPP. However, despite the aforementioned evidence being limited, an attempt was made here to assess the link between the pollution the inhabitants close to a TEPP are exposed to and the population’s respiratory problems. This is just like a retrospective study conducted in New York in 2012, where a positive association was found between the populations that live in neighborhoods near a TEPP and the increase in hospitalizations for asthma, COPD and acute respiratory infections (ARI) at 11.0, 15.0 and 17.0% respectively [6]. Another study in Thailand in 2013 reported that the residents who lived within a 1 km radius of the TEPP had a higher prevalence of allergies (OR = 2.4), asthma (OR = 2.1) and COPD (OR = 2.7) [7]. In Turkey it was verified that forced expiratory volume in one second and forced vital capacity were significantly lower in populations located near a TEPP [8].

In this study, unlike those in the literature, the dependent variable was the consultation rate for BOC in the emergency services of two communes in the province of Concepción. The magnitude of the association was greater as the emergency consultation rates for BOC in the commune with pollution caused by the TEPP were on average 4.9 times higher over 3 years than the location with no pollution caused by a TEPP, which showed a decrease in the RR in the last year, considering that in 2012 the RR was 6.7, which despite being considered favorable does not imply a decrease in the consultation rates for BOC, only a reduction in the differences between the two communes.

Among the independent variables, temperature seemed to be most strongly associated with the consultations rates for BOC in the population. This association was observed in both locations, with *p* < 0.01, with an inverse association and of average intensity, i.e., the lower the temperature the higher the consultation rates for BOC.

The effect of low temperature on the airways has been studied for some time. In pathologies such as asthma, the most convincing hypothesis to date is that cold-induced asthmatic bronchoconstriction is generated by dehydration of the airway mucosa [12], ie temperature would have a direct and independent effect on consultation rates by BOC. This confounding variable does not affect the result of our study since in both communes the temperature did not present statistically significant differences. On the other hand, when the temperature drops, the use of indoor heating increases with methods derived from the combustion of fossil fuels, such as firewood, paraffin or coal. This could explain the interaction between the decrease in temperature and the increase in air pollution.

The remaining variables, with the exception of NO, were associated with the consultation rate for BOC in Coronel but not in Talcahuano, although pollutants such PM_2.5_ and the relative humidity were significantly higher in Talcahuano than in Coronel. These results show that there could be other confounding variables that were not included in the study this time, which would increase the negative effects of the study variables on the consultation rate for BOC in Coronel.

Finally the aim was to identify the effect of living in neighborhoods near a TEPP on the consultation rates for BOC. In this analysis we can indeed corroborate our hypothesis, since the variable place explained a higher percentage of the variance (26.0%), with a β of 0.31; i.e., living near a TEPP increases the consultation rate for BOC. Temperature followed with 18.0% explanation of the variance, with a β of −0.05. Seeking better explanation for the issue, it was decided to create a simple linear regression model only with the weeks with the highest consultation rates, demonstrating that the explanation of the variance of the variable place increased to 28.0%, and was the only confounding variable with a statistically significant *p* value. This would reveal the impact of living in neighborhoods near a TEPP on these rates, even on the temperature variations, which have historically been the main cause of increased consultation for respiratory diseases generally [13]. In the multiple linear regression models, the model that worked best included the variables place and temperature, explaining 44.0% of the variance, which implies that living in Coronel increases the consultation rates for BOC, as does the decrease in temperature.

### Weaknesses of the study

There were various limitations of this study that must be addressed. Since TEPP fed with fossil fuels usually have quite high chimneys, the pollutants can migrate beyond the geographic area that we considered as “exposed”. Thus, the residents in the “clean” area could be exposed to the contamination generated by the TEPP, mainly when the wind is moving in the right direction [8]. We do not have any information on the height of the chimneys of the TEPP and we did not control the variables for wind direction or force.

We also have no data to monitor the potential confounding factors such as genetic predisposition, cigarette smoking, diet and other personal habits that could increase or reduce consultations for BOC. It was also not possible to control for multiple consultations by a single person or for the residents of the study communes who have attended another center not included in the study.

Another important limitation to emphasize is related to the quality of the records obtained from the SINCA. First, we note that all the monitoring stations in Coronel used as formal records by the Chilean Ministry of the Environment are the property of ENDESA and COLBUN; these two companies own the TEPPs in Coronel and which must be monitored and controlled by government entities.

In addition, many monitoring stations began their formal records in 2013 and 2014. In some cases, there were entire months and even years with no records; therefore, it was not possible to work with these stations. Additionally, some of the environmental pollutants described by the evidence as harmful to the respiratory system were not even recorded, such as NO_2_ and SO_2_.

Specifically in the commune of Talcahuano, none of the monitoring centers had meteorological records, thus it was necessary to contact the Chilean Navy directly to obtain this data. On this same point, the north Coronel monitoring station is located more than 3 km from the coast, unlike the navy monitoring station in Talcahuano, which is located on the shoreline and much more exposed to humidity from the sea. It is evident that any alteration in the recorded data could cause significant changes in the results.

Another final limitation is the existence of ecological fallacy, which appears when results are generalized to the entire population living in the geographic areas included in this study.

Although ecological studies like this must always be considered hypothesis-generating, the consistency between these results and those reported in the literature provide additional evidence that the use of publicly available data has value in the identification of disease patterns in relation to residential exposure. This study quantifies differences in BOC rates for people living near a coal-fired TEEP compared to those living 40 km away, and explores potential predictors for the observed variation.

## Conclusions

Living near a coal-fired TEPP is associated with an increased risk of emergency consultation for BOC in the study population.

## Abbreviations

BOC: Bronchial obstructive crises
CESFAM: Family Health Centers
CO: Carbon monoxide
CO2: Carbon dioxide
COPD: Chronic obstructive pulmonary disease
CRD: Chronic respiratory diseases
DAS: Office of Health Administration
DEIS: Office of Health Statistics and Information
ICD-10: International Classification of Diseases
NO: Nitrogen oxide
PM: Particulate matter
SAPU: Emergency primary care units
SINCA: National Air Quality System
SO2: Sulfur dioxide
TEPP: Thermoelectric power plants
